# Modified Kraske Procedure with Mid-Sacrectomy and Coccygectomy for *En Bloc* Excision of Sacral Giant Cell Tumors

**DOI:** 10.1155/2014/834537

**Published:** 2014-10-16

**Authors:** Vítor M. Gonçalves, Álvaro Lima, João Gíria, Nuno Carvalho, José Parreira, Manuel Cunha e Sá

**Affiliations:** ^1^Neurosurgery Department, Garcia de Orta Hospital, Avenida Torrado da Silva, 2801-951 Almada, Portugal; ^2^Orthopedic Department, Beatriz Ângelo Hospital, Avenida Carlos Teixeira, 2674-514 Loures, Portugal; ^3^General Surgery Department, CUF Infante Santo Hospital, Travessa do Castro, 1350-070 Lisbon, Portugal; ^4^General Surgery Department, Garcia de Orta Hospital, Avenida Torrado da Silva, 2801-951 Almada, Portugal; ^5^Plastic and Reconstructive Surgery Department, Garcia de Orta Hospital, Avenida Torrado da Silva, 2801-951 Almada, Portugal

## Abstract

Sacral giant cell tumors are rare neoplasms, histologically benign but potentially very aggressive due to the difficulty in achieving a complete resection, their high recurrence rate, and metastization capability. Although many treatment options have been proposed, *en bloc* excision with tumor-free margins seems to be the most effective, being associated with long term tumor control, improved outcome, and potential cure. An exemplifying case of a 29-year-old female with progressive complaints of pain and paresthesias in the sacral and perianal regions, constipation, and weight loss for 6 months is presented. The surgical technique for *en bloc* excision of a large sacral giant cell tumor through a modified Kraske procedure with mid-sacrectomy and coccygectomy is described. Complete resection with wide tumor-free margins was achieved. At 5 years of follow-up the patient is neurologically intact, without evidence of local recurrence on imaging studies. A multidisciplinary surgical procedure is mandatory to completely remove sacral tumors. In the particular case of giant cell tumors, it allows minimizing local recurrence preserving neurovascular function, through a single dorsal and definitive approach.

## 1. Introduction

Giant cell tumors (GCT) of bone are rare neoplasms comprising 5% of all primary bone tumors in adults [1] and 5 to 10% of all benign bone tumors [2], with a 2% to 8.2% incidence rate [3–5]. They usually affect metaepiphyseal regions of long bones, most often in the knee and radius. Sacrum is the third most common site of involvement [2] and the most affected bone of the axial skeleton, accounting for 2–8% of all GCT [6–8]. This type of neoplasm is the second most frequent primary bone-involved tumor in the sacrum [4].

GCT are histologically benign, presenting a slow growth rate and insidious or clinically silent onset, making early diagnosis difficult. Usually they exhibit a very large size when diagnosis is made [8]. They are locally highly aggressive and present a high recurrence rate and the power to metastasize, being associated with high morbidity [2, 9–13]. Although considered benign, they are usually lethal, making them a complex medical disease [14–16]. Distant metastization is unusual. The reported incidence of lung metastases from a histologically proven GCT ranges from 1% to 9% [9, 17–20]. The local recurrence rate seems to be as high as 33% [4], reaching more than 50% when intralesional curettage excision is performed [2, 8]. This may be explained by difficulties in achieving an early diagnosis, the large tumor volume at initial presentation, aggressive behavior, poorly defined tumor margins, and the difficulty to surgically access these lesions without harming the patient [1, 5, 20]. Local malignant transformation has also been reported, accounting for 16% of primary cases [8, 21].

Magnetic resonance imaging (MRI) and computed tomography (CT) scans are useful for early diagnosis and preoperative planning [22]. Needle biopsy may be reserved for selected cases [23, 24].

Different treatment options have been used for sacral GCT [7, 14]. These tumors are relatively resistant to radiation therapy [4, 14, 15, 17], which on the long term may result in radiation-induced sarcoma (3–11%) [15, 21, 25, 26]; no standard chemotherapy protocols are available. This may be the reason why such treatment options remain controversial [14, 25].

When located in the sacrum, surgical resection is the primary treatment modality, being advocated by most authors [4, 5, 27–29].* En bloc *excision with tumor-free margins, although challenging, is the procedure of choice, once this constitutes the most effective method for local disease control and recurrence prevention, improving outcome and providing the best chance for cure [3, 4, 14, 17, 28].

We present an exemplifying case of a patient harboring a lesion, which was surgically treated through a modified Kraske procedure with mid-sacrectomy and coccygectomy, for* en bloc* excision of the tumor, with wide tumor-free margins. A detailed and comprehensive step-by-step surgical technique overview is presented.

## 2. Case Presentation

A 29-year-old female, without known past medical history, was admitted with progressive complaints of severe pain and paresthesias in the sacral and perianal regionsfor 6 months. In this period of time she also presented constipation and 5 Kg weight loss. These symptoms were refractory to medical therapy. Pain exacerbated in the night and by Valsalva maneuvers, causing severe functional disability.

Physical examination revealed severe pain on palpation and percussion of the sacral region, without a visible or palpable lesion or other signs of inflammation. Digital rectal examination revealed a large midline presacral mass, fixed to the sacrum, with firm consistency and irregular surface.

**Figure 1:**
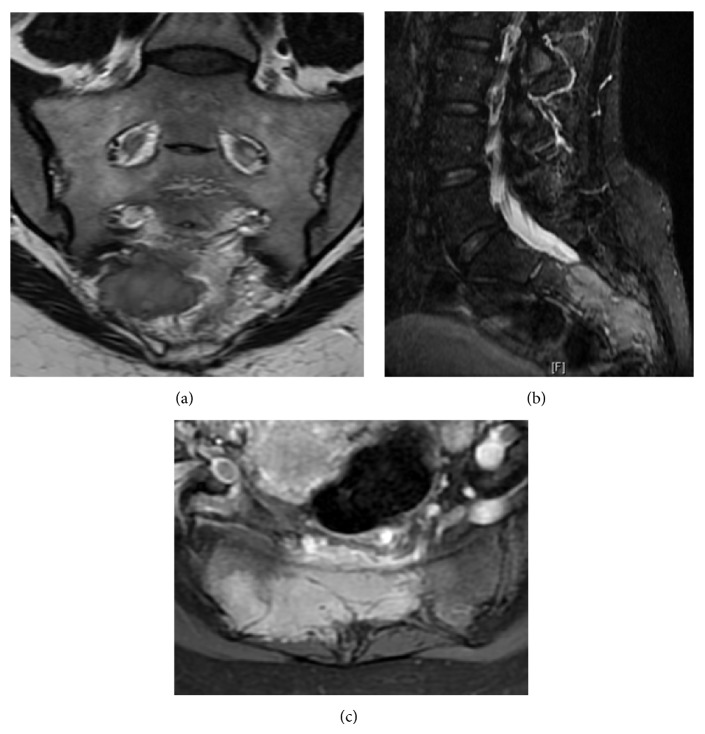
Preoperative T2-weighted contrast enhanced MRI showing an expansive and osteolytic lower and mid-sacral lesion, extending up to the inferior half of S2 vertebra, with both intra- and extracanalar components and a ventral extension displacing the rectum anteriorly. (a) Coronal, (b) sagittal, and (c) axial views.

The lumbosacral CT and MRI scans showed a large, expansive, and osteolytic lower and mid-sacral lesion, with poorly defined margins, extending up to the inferior half of S2 vertebra. The mass comprised both intra- and extracanalar components, a ventral extension displacing the rectum anteriorly, and dorsal expansion out of the sacral hiatus and dorsal foramina with soft tissue compromise. It was located in the midline, slightly more pronounced on the right side, in between the inferior half of S2 vertebra and the sacrococcygeal junction. S2 nerve roots were spared but all nerve roots distal to that were involved by the tumor. The coccyx was not affected (Figure 1).

Further diagnostic workup was performed including laboratory studies with tumor markers, chest, abdomen, and pelvis CT-scan and positron emission tomography. No abnormalities or other lesions were detected.

After complete characterization of the boundaries of this sacral solitary lesion, a multidisciplinary elective and definitive surgery was scheduled with the collaboration of general and plastic surgeons. No previous biopsy was performed.

### 2.1. Operative Technique

**Figure 2:**
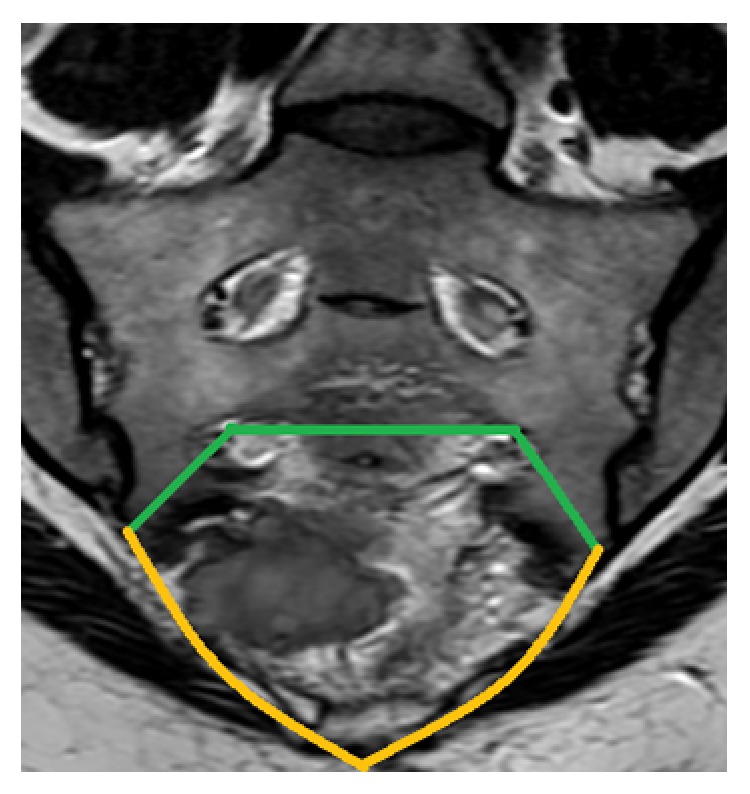
Previewed* en bloc* excision of the tumor on coronal MRI. Green and yellow lines represent the limits of the specimen to be resected, respectively, corresponding to the 3 sacral osteotomies and the inferior margin around the tumor.

The patient was electively operated by one of the senior authors (A.L.).* En bloc* excision of the tumor with wide tumor-free margins through a modified Kraske procedure with mid-sacrectomy and coccygectomy was achieved requiring sacrifice of the nerve roots and thecal sac below the level of S2 nerve roots (Figure 2).

*Anesthesia and Positioning.* Under general anesthesia the patient was intubated. Arterial line was placed for blood pressure monitoring. Intravenous dexamethasone and antibiotic prophylaxis (cefazolin 1 g) were administered preoperatively. The patient was positioned prone. All pressure points were covered with padding. Care was taken to avoid elevated abdominal and airway pressures because this would lead to inconvenient bleeding. The posterior lumbosacral area, low back buttock, and posterior thighs were subsequently sterilized and draped after the skin was dried, giving the plastic surgeons many options for soft tissue reconstruction and wound closure at the final step of the surgery. Extra care was taken at this stage due to the proximity of the anal orifice to the surgical field, increasing the risk of wound contamination.

**Figure 3:**
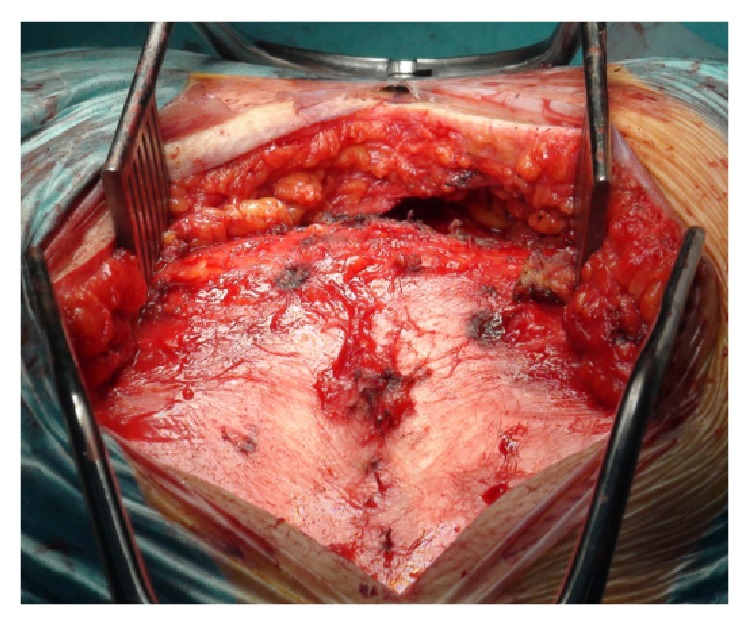
Exposition of dorsal fascia from lumbosacral junction to the tip of the coccyx.

*Modified Kraske Procedure.* A midline longitudinal skin incision was carried out posteriorly extending from the lumbosacral junction to the coccyx. The sacral fascia was exposed from L5-S1 level to the tip of the coccyx (Figure 3).

**Figure 4:**
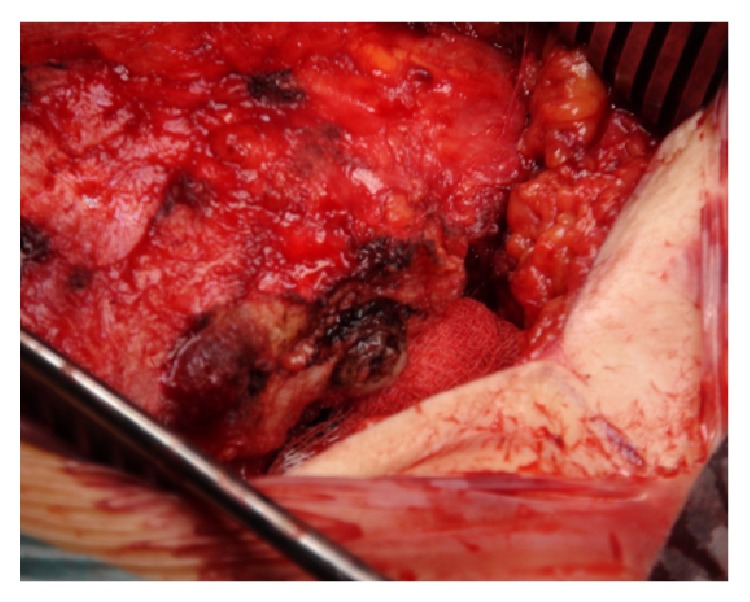
Inferior soft tissue dissection exposing the tip of the coccyx.

These limits were, respectively, superior and inferior to the tumor and not involved by it. Initially, the fascia opening and soft tissue dissection were performed inferiorly exposing the tip of the coccyx and then coming around anterior to it (Figure 4).

The anococcygeal ligament was transected at a distance from the anal sphincter and, working ventral to the coccyx, the levator ani muscles were detached from it and retracted laterally allowing the approach to the presacral space.

**Figure 5:**
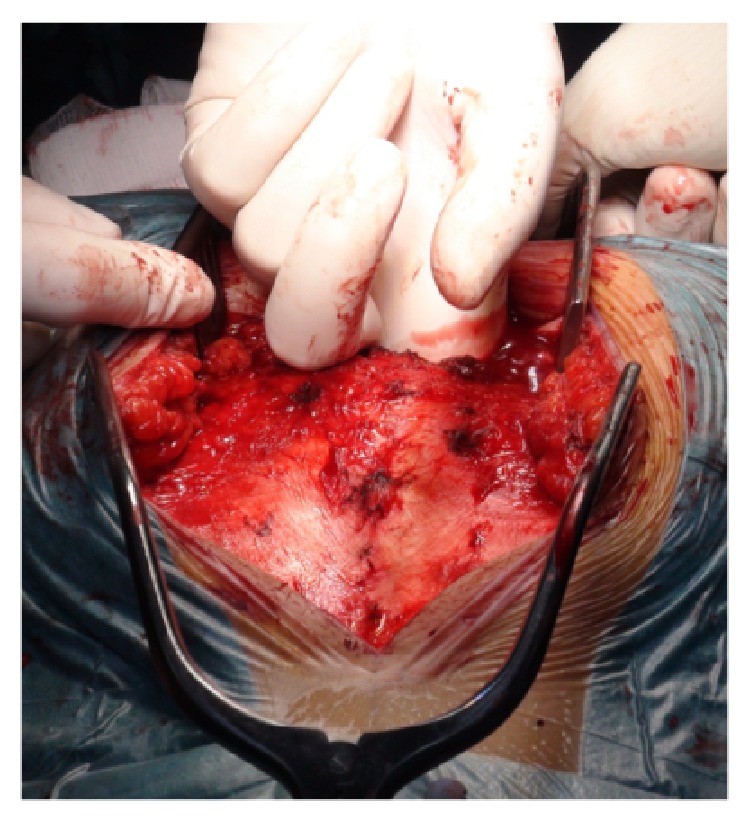
Initial finger dissection used to mobilize the rectum.

Kraske approach was then performed. Finger dissection was used to mobilize the rectum creating a plane between the posterior aspect of the rectum and the ventral part of the sacrum including the anterior surface of the tumor (Figure 5).

**Figure 6:**
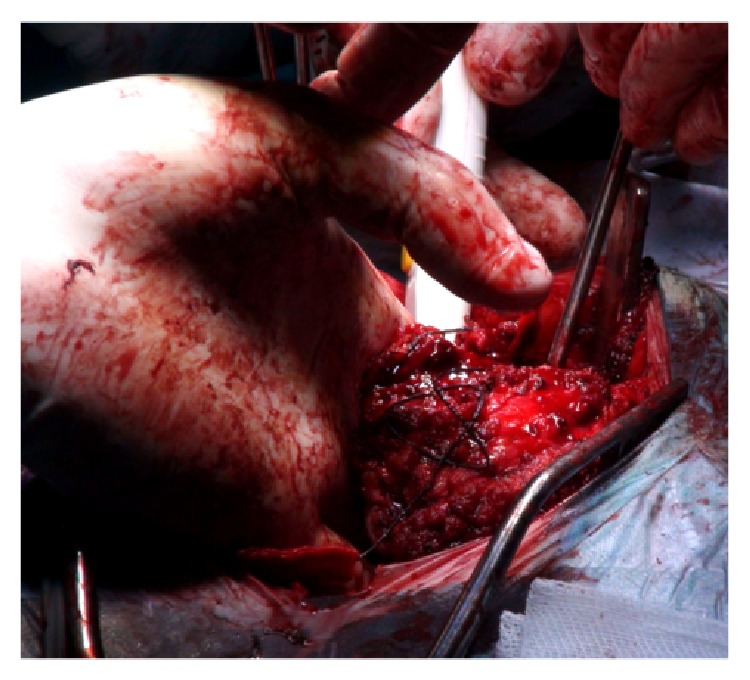
Kraske procedure providing access between mesorectum and presacral component of the tumor.

Once this was accomplished, a coccygectomy was executed to provide a better exposure. At this stage, general surgeons help dissecting Waldeyer's fasciacreating an avascular plan between the mesorectal fascia propria and the presacral fascia. This approach provided access between the mesorectum and the presacral component of the tumor, up to its superior portion at the level of the inferior half of S2 segment. Additional release of the lateral ligamentous attachments and the inferior portion of the gluteal muscles allowed a full hand to be insinuated into this plane and provide further dissection and palpation of the sacrum above the tumor (Figure 6).

Creation of this space was very important to protect the rectum and provide tactile feedback and guidance during the subsequent osteotomies. The obtained dissection plane was conveniently preserved with large surgical patties with identification string.

**Figure 7:**
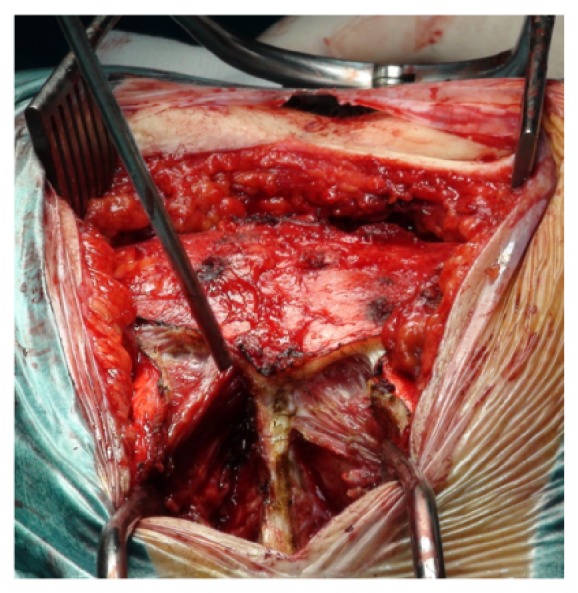
Superior subperiosteal dissection.

Fascia opening and subperiosteal dissection were then performed superiorly, exposing the tumor-free dorsal surface of the upper sacrum at the S1 and S2 levels (Figure 7).

**Figure 8:**
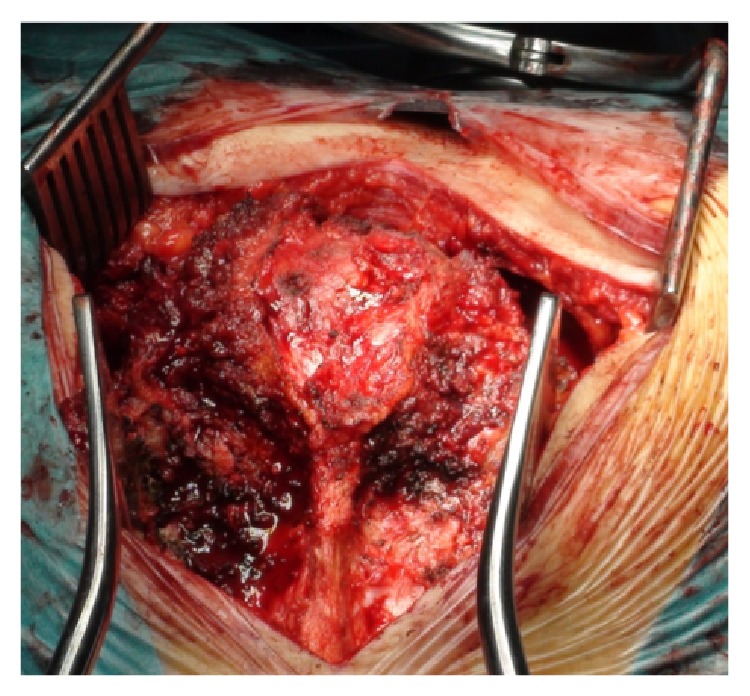
Soft tissues intentionally left behind and included in the specimen to be resected.

Lower to this level, the sacral periosteum was not incised or dissected. There, the paraspinal muscles were truncated leaving an island of sacrospinalis musculature and fat overlying the dorsal surface of the sacrum, in an attempt to leave a tumor-free margin, due to the tumor infiltration posteriorly through the dorsal foramina and sacral hiatus. The distal portion of these muscles remained attached to the sacral specimen being removed together afterwards (Figure 8).

Laterally at the distal sacrum, the gluteus muscle attachments and the sacrospinous and sacrotuberous ligaments were detached exposing the coccygeus and piriformis muscles, which were divided revealing the lower elements of the sacral plexus. Subperiosteal dissection was subsequently carried out over the posterior superior iliac spines allowing mobilizing the soft tissues to bilaterally expose the sciatic notches.

At this point the Kraske approach was performed inferiorly. Superiorly we have exposed the dorsal bony elements including the S1 and S2 lamina, as well as posterior superior iliac spines.

*Laminectomy*,* Thecal Sac*,* and Nerve Root Ligation.* Under 2,5x magnification surgical loupes view, S1 and S2 laminectomies were accomplished using a fine Kerrison* rongeur*, allowing exposition of the thecal sac and the tumor-free S1 and S2 nerve roots, going to the respective foramina. At this point it was very important to make sure the correct level was identified, not to harm the inappropriate nerve roots, as the thecal sac was going to be ligated. S1 and S2 nerve roots were correctly identified. The S2 nerve roots were dissected and skeletonized on their way to the respective foramen. Tumor was identified bilaterally in the axilla of the S3 nerve roots which were too intimately involved by the tumor to be spared. Tumor capsule was intact.

**Figure 9:**
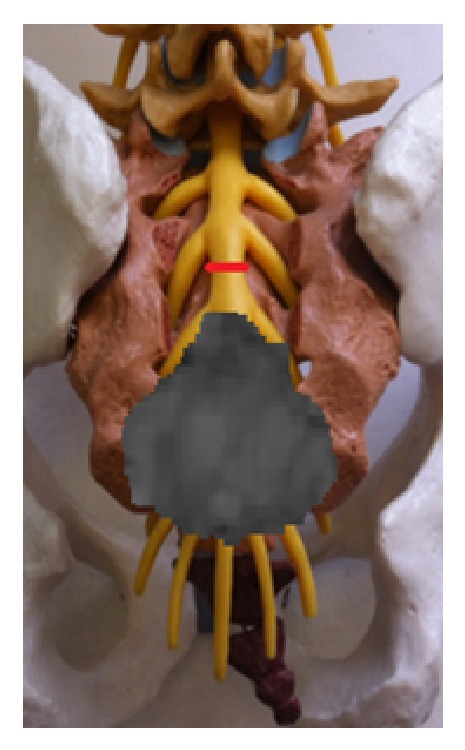
Thecal sac ligation distal to S2 nerve roots (red line).

Thecal sac ligation was then performed with two 2-0 silk ties passed around the thecal sac, distal to S2 nerve roots (Figure 9).

A scalpel blade number 15 was then used to sharply cut the thecal sac, distal to these ties. The distal thecal sac and S3, S4, and S5 nerve roots were compromised by the tumor, being sacrificed and included within the specimen to amputate. A meticulous hemostasis was achieved with coagulation of epidural venous plexus.

**Figure 10:**
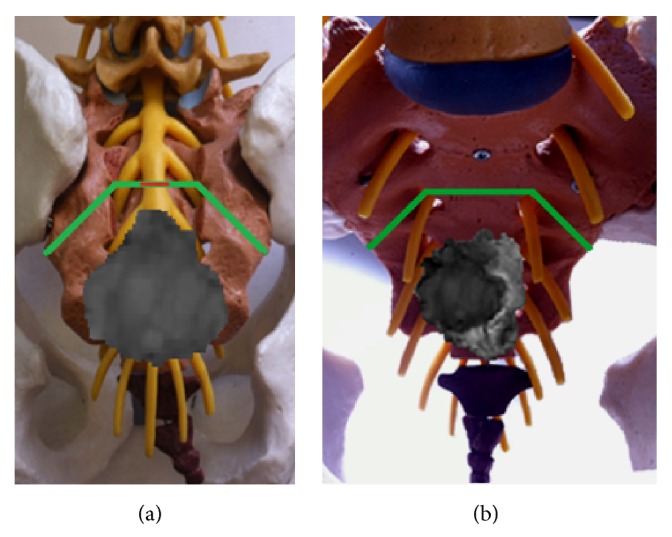
Representation in a model of the 3 osteotomies performed (green lines) and the level of thecal sac ligation (thin red line). (a) Posterior view and (b) anterior view.

*Sacral Osteotomies.* Three sacral osteotomies were performed, two oblique, executed laterally on each side, between the S2 foramen and the ipsilateral greater sciatic notch, followed by a medial transverse osteotomy, done between the S2 foramina (Figure 10).

After thecal sac and nerve root ligation was achieved, bilateral S2 nerve roots were extensively dissected and followed on their way to the respective S2 foramina. This constitutes an important landmark for the execution of lateral osteotomies, once these extend between the S2 foramina laterally and the greater sciatic notch. Remaining soft tissues at the sciatic notch were dissected with monopolar electrocautery, enabling a finger to be insinuated superior to the piriformis muscle into the sciatic notch for further dissection, and advanced medially to palpate the ventral S2 foramen. With the finger in place, this important maneuver allowed guiding and safely performing the lateral osteotomies. Once the osteotomy has passed from the S2 foramen out to the sciatic notch, the lateral aspect of the sacrum was cut allowing the lateral osteotomies to be completed. This step was accomplished bilaterally. Sacroiliac joints were completely spared and safeguarded.

Having completed the lateral osteotomies, some additional dorsal gluteal musculature was taken down laterally with the monopolar electrocautery. The Kraske approach was again used for guidance of the transverse osteotomy. The tactile feedback helped to direct the bone cut. This could be performed with osteotomes oriented in a transverse direction between the S2 foramina, beginning at one S2 foramen and carrying over to the other. The hand inserted by the Kraske approach into the presacral space protected the dorsal aspect of the rectum and again provided additional tactile feedback for the osteotomy. It helped to guide osteotomes' trajectory and sense when the anterior bony cortex was perforated and bony cut has been completed. Bleeding from the sacral osteotomies was controlled with bone wax.

*En Bloc Resection of the Tumor.* At this point, we performed a coccygectomy and the Kraske approach inferiorly, gluteal musculature release laterally, thecal sac and nerve root ligation below the level of S2 nerve roots, and lateral and transverse osteotomies superiorly. After this was accomplished, the specimen was tilted dorsally, stretching the S2 nerve roots so that they could be extensively dissected all the way from their origin at the thecal sac, freeing them from the remaining foramina and tracing them out distally.

With additional mobilization, soft tissue attachments were subsequently released. The remaining deeper muscular and ligamentous (sacrotuberous and sacrospinous ligaments) attachments and the distal ends of the S3, S4, and S5 nerve roots were identified and cut with the monopolar electrocautery. This allowed the specimen to be circumferentially freed and removed from the surgical field, resulting in a satisfactory* en bloc* resection with wide tumor-free margins.

**Figure 11:**
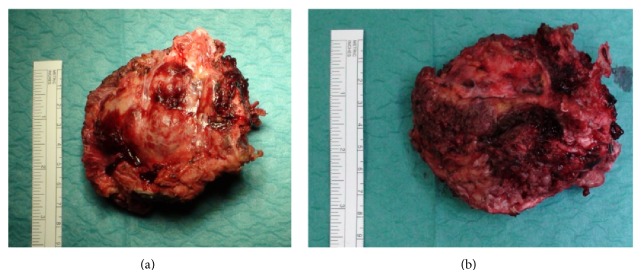
*En bloc* resected specimen. (a) Ventral surface and (b) dorsal surface.

**Figure 12:**
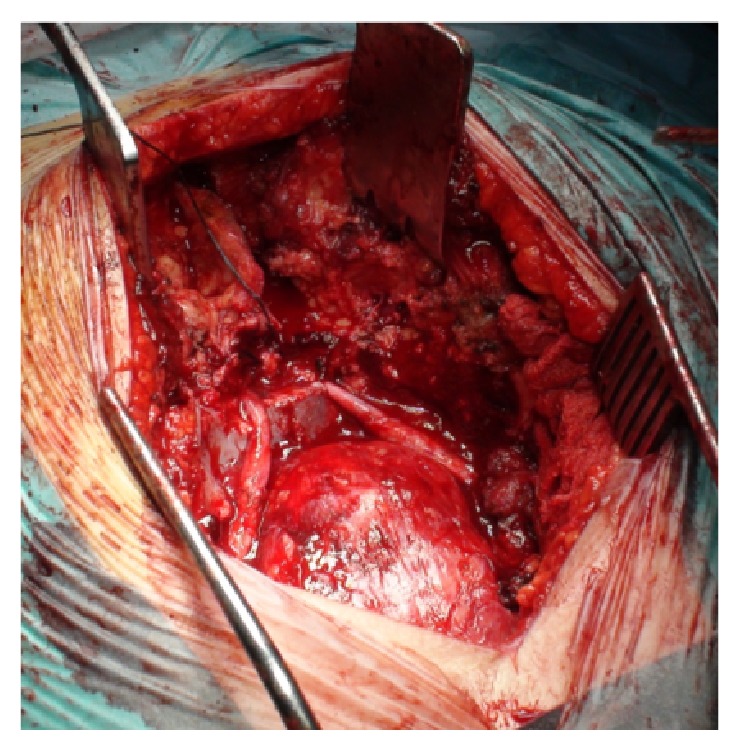
S2 nerve roots were preserved and remained intact along their entire length.

The specimen included the tumor with its presacral component (Figure 11(a)) and the dorsal paraspinal muscles, left to provide a wide margin posteriorly (Figure 11(b)). A satisfactory superior margin was also achieved. With this technique the tumor capsule was not disrupted and S2 nerve roots were preserved and remained intact along their entire length (Figure 12). 

*Hemostasis and Closure.* A large dead-space cavity resulted from the excision of the specimen. Careful hemostasis of the presacral soft tissue was achieved. Bleeding from the sacral osteotomies was controlled with bone wax.

**Figure 13:**
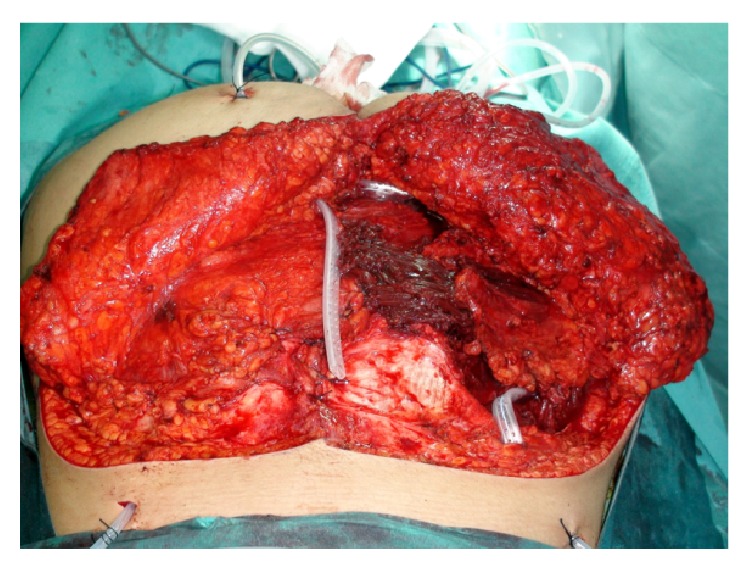
Rotational flaps of the gluteus maximus provided for soft tissue reconstruction and wound closure.

General surgeons helped reapproximating the levator ani muscles. In the final step of the surgery, plastic surgeons provided soft tissue reconstruction to fill the defect and close the wound. At this time, the skin incision was lengthened incorporating the prior midline incision. Adipomuscular mobilization and rotational flaps of the gluteus maximus were used in the reconstruction of the sacral defect (Figure 13). Adequate soft tissue reconstruction was achieved, as well as wound closure in a layered and tensionless fashion. Two suction drains were left in place. Cefazolin was used in prophylactic dose (1 gm IV) 60 minutes before surgery and then every six hours during 24 hours postoperatively.

## 3. Results 

*En bloc* excision with wide tumor-free margins of this large lower and mid-sacral mass was achieved through a modified Kraske procedure with mid-sacrectomy and coccygectomy. It required sacrifice of the nerve roots and thecal sac below the level of S2 nerve roots.

As previewed on the preoperative MRI scan, the tumor location at the lower and mid-sacrum and its limits (superiorly: the inferior half of S2 segment; inferiorly: the superior half of S5 segment; laterally: sparing the sacroiliac joints; ventrally: in intimate relation with the rectum which was anteriorly displaced; dorsally: invading the posterior surface of the sacrum out through the sacral hiatus and dorsal foramina; inside the sacral canal: affecting the thecal sac and nerve roots below the S2 nerve roots level) were corroborated with the intraoperative findings and allowed to precisely define the boundaries of the specimen to be removed, keeping distance from the tumor capsule and sacroiliac joints.

**Figure 14:**
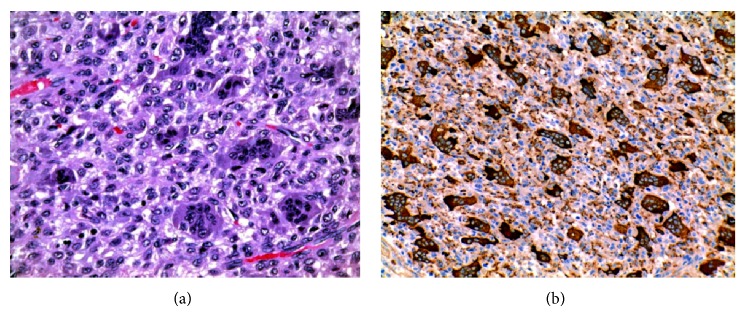
(a) Hematoxylin-eosin stained tissue demonstrated a highly cellular, solid neoplasm consisting of mononuclear cells and osteoclast-like giant cells; (b) intense immunohistochemical staining for CD68 (KP1).

There were no procedure-related complications. Histopathological analysis of the resected specimen revealed a benign GCT (Figure 14).

**Figure 15:**
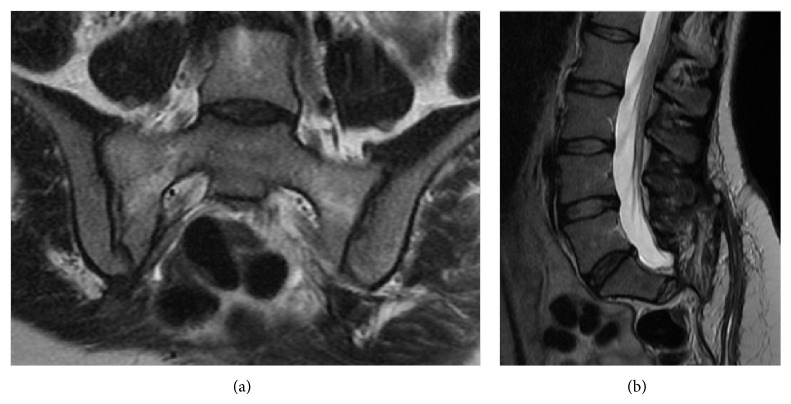
MRI scan evaluation 5 years after surgery, showing no recurrence of the tumor. (a) Coronal and (b) sagittal views.

The patient was discharged home presenting constipation and urinary retention. Constipation resolved with laxatives. Concerning urinary retention she underwent a suitable rehabilitation program, with intermittent self-catheterization, and perineal muscular tonification workout with voluntary sphincter contraction exercises. This resolved within five months following surgery. At five years of follow-up, the patient still complains of minimal subjective numbness in the perianal region, without any other neurological deficits and performing well in all living activities, being inclusively able to sit for long periods of time without pain. The postoperative MRI scan showed complete resection of the distal sacrum and coccyx with no evidence of residual lesion. Last follow-up MRI was performed five years after surgery showing no recurrence of the tumor (Figure 15).

## 4. Discussion

Management of GCT of the sacrum is complex and challenging from diagnosis to treatment. This is due to their rarity and heterogeneous clinical scenarios and because the surgical procedures generally involved are extensive and aggressive, aiming for complete tumor resection and potential cure.

These procedures may be extra-demanding not only because of the aggressive nature and behavior of GCT, but also once the majority of them are diagnosed in an advanced stage of the disease, exhibiting large volumes and sometimes, poorly defined margins. This may create technical difficulties in surgical access and tumor resection, due to the surrounding anatomical constraints and nearby noble structures to preserve [1, 5, 20, 30]. For this reason, proper patient selection is paramount.

A detailed clinical and neurological assessment including digital rectal examination is mandatory for helping to establish an early diagnosis. Investigation for metastatic disease should always be performed in order to decide the best treatment option (curative versus palliative).

Tumors' radiological appearance and their location, accessibility, local extension, and involvement of adjacent neurovascular structures are of paramount importance and should always be kept in mind before considering performing a biopsy, once this is an invasive, noninnocuous procedure, with well-known risks of tissue contamination through the biopsy tract, hemorrhage, and infection [14, 31]. CT-guided fine-needle biopsy may be a helpful tool for the histological diagnosis [14] in selected cases: whenever this information is preoperatively relevant for potentially changing or influencing the treatment approach; for unresectable lesions; or in patients with significant comorbidity, precluding a more aggressive surgery (if required to indicate adjuvant or palliative therapy). Biopsy is useful to reach the differential diagnosis of sacral lesions (metastases, giant cell tumor, chordoma, teratoma, and chondrosarcoma). In this particular case, we adopted a direct approach to the lesion, without previous biopsy, because the patient had a good general medical condition; there were no other documented lesions; the tumor was well circumscribed, surgically accessible with the possibility of reaching* en bloc* resection with tumor free margins without further neurological deterioration. Having this in mind, the surgical approach would be the same regardless of the biopsy result.

Preoperatively, it is important to precisely define the goal of surgery and the anatomical boundaries to be respected intraoperatively. It is crucial to accurately assess several imagiological parameters such as the level of sacral involvement, infiltrated structures (sacral canal, thecal sac, nerve roots, muscles, ligaments, vascular and visceral structures, and sacroiliac joints), and ventral and dorsal extensions. This is essential to establish a preliminary diagnosis and decide the best surgical approach and for surgical planification of the* en bloc* resection [22].

Several treatment options have been proposed: intratumoral curettage plus radiotherapy, possibly aided by preoperative embolization, the use of osteoclast inhibiting drugs (bisphosphonates), and cryosurgery with liquid nitrogen, limited by the risk of injury to adjacent neural structures in sacral tumors.

According to some authors,* en bloc* excision is the gold standard procedure for sacral GCT which present radiological criteria denoting the potential aggressiveness of the tumor: poorly defined margins, cortical bone destruction, and soft tissue extension by expansive tumor growth [6, 30, 32–34].

The presented surgical technique is an optimal and effective strategy to completely remove lower and mid-sacral tumors. It is indicated for the benign but highly aggressive sacral GCT and can also be applied to primary malignant bone tumors of the sacrum. In the particular case of GCT, the modified Kraske procedure with mid-sacrectomy and coccygectomy allows maximizing local tumor control, minimizing the risk of local recurrence, and providing possible cure, giving the possibility to preserve neurovascular function, by this mean decreasing morbidity and improving the final outcome.

During the surgery of lower or mid-sacral tumors, several nuances must be taken into account. Only the affected sacral nerve roots should be sacrificed, and the expected neurological outcome needs to be preoperatively predicted and discussed with the patient [33, 35–37]. Also, the sacroiliac joints should be spared to avoid spinal instability [38–42].

Given the complexity of evaluation, treatment, and management of GCT, a coordinated multidisciplinary team approach to the problem, involving neurosurgeons, general surgeons, and plastic surgeons, working together in specialized units, has proved useful [33]. This collaboration is essential helping to select and implement surgical treatment to minimize the risk of perioperative complications. General surgeons are important for the approach and mobilization of the rectum from the tumor and ventral sacrum.

The used modified Kraske procedure (midline incision rather than left parasacral as it was described in the original technique) provided an excellent surgical access between the mesorectum and the presacral component of the tumor, allowing a safe and complete tumor removal to be achieved without morbidity [31, 43]. Also plastic surgeons collaboration is highly significant for soft tissue reconstruction and wound closure. The extensive resection of sacral tumors is always associated with large defects or dead-space cavities, and to optimize surgical wound outcome, minimizing the risk of wound dehiscence or infection, their intervention is crucial [44–48]. We adopted the closure technique described by Yao et al. [49]. This technique has many advantages over other reconstruction and closure approaches: it keeps a native and robust blood supply of the flaps, creates a protective tissue layer while also absorbing cavity effusion, and reduces seroma formation, wound dehiscence, and rate of infection, which can reach as much as 38% [49].

The major benefits of this surgical technique are the capability to successfully achieve complete surgical resection with wide tumor-free margins, protect the presacral vascular and visceral structures, and accomplish these goals through a single and definitive dorsal approach. Trying to accomplish complete removal of GCT during the initial surgery is very important and should be, if possible, the main goal. This allows reaching a favorable prognosis, minimizing the risk of recurrence [28]. Nevertheless, careful selection of the patients amenable to this approach is a must. Low or mid-sacral tumors constitute the perfect indication.

## 5. Conclusion

Surgical treatment of sacral GCT is challenging and technically demanding, due to the complex regional anatomy in this area, and the advanced stage of disease by the time diagnosis is made. A well-coordinated multidisciplinary team approach, working in specialized units, is mandatory.

Early diagnosis, complete (*en bloc*) surgical resection with tumor-free margins, and a comprehensive treatment are essential for local tumor control, best long-term prognosis, and improved outcome with possible cure.

The difficult conflict between patient's functional integrity and the cure of the disease must be preoperatively well weighted and discussed with the patients and the team.

An accurate preoperative planning must precisely locate the tissues involvement (bone, muscle, nerves, and joints) and delineate the extension of the area to be resected.
